# Management Strategies for Dealing With Surges of the COVID-19 Pandemic

**DOI:** 10.7759/cureus.15794

**Published:** 2021-06-21

**Authors:** A.V. Raveendran, Jothydev Kesavadev, Parameswaran Hari, Gopika Krishnan

**Affiliations:** 1 Internal Medicine, Government Medical College, Kozhikode, IND; 2 Internal Medicine, Badr Al Samaa Hospital, Barka, OMN; 3 Diabetes and Endocrinology, Jothydev’s Diabetes Research Centre, Thiruvananthapuram, IND; 4 Hematology and Oncology, Medical College of Wisconsin, Wauwatosa, USA

**Keywords:** covid-19, sars-cov-2, pandemic, management, home treatment

## Abstract

The spread of COVID-19 (coronavirus disease 2019) across the world has resulted in widespread morbidity and mortality. An explosive increase in the number of cases during the surge phase of the pandemic can result in a management crisis. Therefore, we propose a simple model to manage the surges of the pandemic.

## Introduction

SARS-CoV-2 (severe acute respiratory syndrome coronavirus 2) infection, which started in Wuhan, China has by now spread all over the world causing widespread morbidity and mortality. An explosive increase in the number of cases occurs during the surges of the pandemic when even developed countries fail to accommodate and manage it properly. In addition to the shortage of hospital beds, ICU beds, ventilators, oxygen, and essential drugs, even the number of health care providers becomes insufficient to provide care in most situations of explosive spread. During the peak of the pandemic, similar crises have been reported from all parts of the world. To tide over the crisis, health care workers are forced to work continuously and still fail to address the issues. Proper planning and anticipation can help to utilize the resources more efficiently thereby enabling the provision of appropriate care to a larger number of citizens.

## Technical report

As of 17 May 2021, the total number of cases of COVID-19 (coronavirus disease 2019) in India is around 25,227,970 and around 3,50,000 new cases are being added to this daily during the peak of the ongoing second wave [1]. Patients with a severe disease along with hypoxia require ventilators for an average of three to six days which can even be extended for weeks and to top this, new critical cases are also added every day resulting in a severe shortage of ventilators, oxygen supply, essential medicines, and hospital beds. However, only about 1.9 million hospital beds, 95,000 ICU beds, and 48,000 ventilators are available in India to fight against this crisis [2].

**Figure 1:**
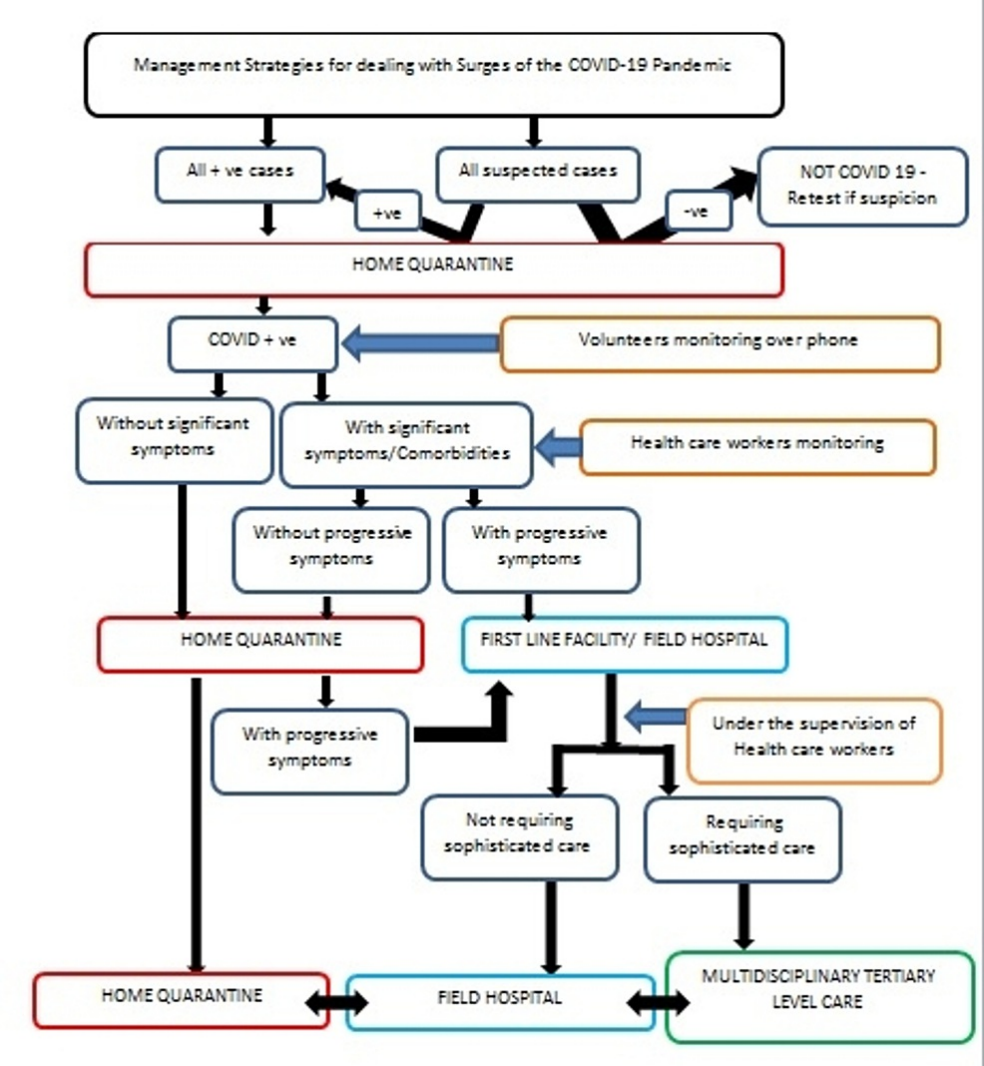
Proposed model to manage the surges of the pandemic

We propose a simple model to manage a pandemic surge, as even a well-established health system may fail to accommodate a large number of cases (Figure 1 and Table 1).

**Table 1:** Monitoring and treatment options of COVID-19 at different levels H2 blocker: Histamine-2 receptor blocker; PPI: Proton pump inhibitor; COVID-19: Coronavirus disease 2019.

Symptoms	Monitoring	Treatment
At-home quarantine		
Fever	Temperature	Paracetamol
Body ache		Anti-histamines
Headache	Pulse rate	Antibiotics
Running nose		Cough syrup
Cough	Respiratory rate	H2 blockers/PPI
Throat pain		Steroids
Breathlessness	Blood pressure	Anti-coagulants
Abdominal discomfort		
Diarrhea	Oxygen saturation (SpO2)	
Any co-morbidities	Blood glucose with glucometer	Optimization of treatment of co-morbidities
	Full blood count, inflammatory markers, and biochemical panels if healthcare workers visit	
At first line facility/ field hospital		
Breathlessness	All of the above	All of the above
	Chest X-ray	Inj. Dexamethasone
	Routine blood test including inflammatory markers and biochemical panels	Monoclonal antibody- Inj. Tocilizumab
		Inj. Heparin
		Oxygen
Deterioration of comorbid condition	Monitoring of comorbidities	Optimization of treatment of comorbidities
At multidisciplinary tertiary level care		
Breathlessness even at maximal oxygen supplementation	All of the above	All of the above
	Investigations as per patients profile	Ventilator support
Deterioration of comorbid condition	Monitoring of comorbidities	Optimization of treatment of comorbidities
Early identification of complications	Monitoring of complications	Treatment of complications

## Discussion

**Figure 2:**
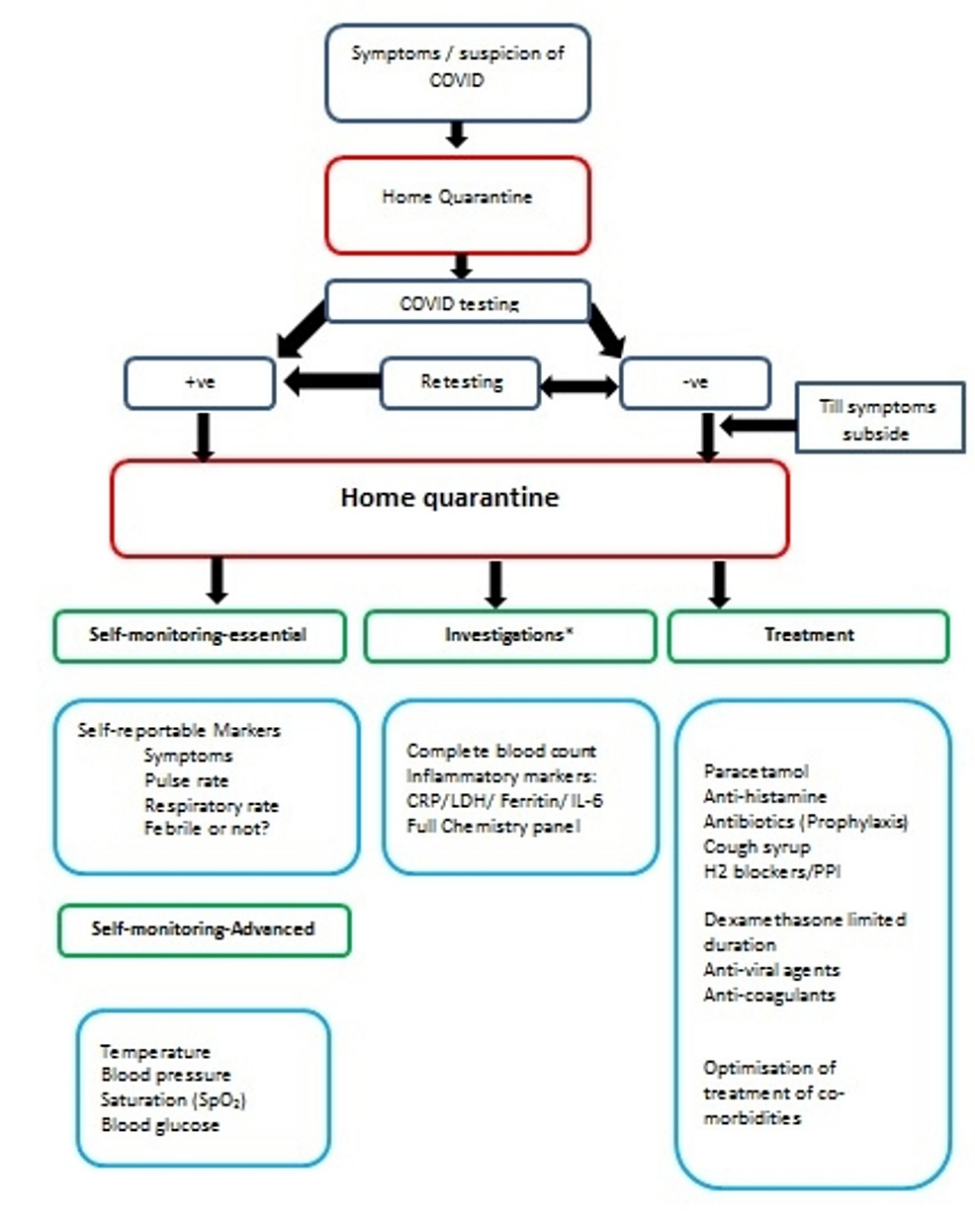
Treatment options during home quarentine

The majority of people affected with COVID-19 develop only asymptomatic or mildly symptomatic disease [3, 4]. Those who develop mild COVID-19 should be monitored and treated at home by trained family members or volunteers, with intermittent support from healthcare providers. The latter may recommend simple treatment measures like paracetamol, antihistamines, cough syrup, etc (Figure 2) [5]. Managing asymptomatic and uncomplicated cases (which constitute the majority of the cases during this pandemic) at home is the key step in the success of the management strategy.

Those who have significant symptoms or associated co-morbidities should be monitored more closely by trained paramedical staff under the supervision of a doctor. The latter may administer drugs like antibiotics, anticoagulants, steroids, etc., depending upon the clinical scenario. Finally, those with moderate-to-severe COVID-19 or deteriorating symptoms such as hypoxia requiring oxygen should be shifted to hospitals, where volunteers, paramedical staff, and doctors are at hand to take care of these patients. Those developing hypoxia despite oxygen support may require positive-pressure ventilation support or multi-disciplinary interventions. Therefore, they should be shifted to tertiary-care facilities. We propose that this strategy will reduce unnecessary hospital admissions and resource utilization, thereby helping appropriate allocation of scarce resources to needy patients.

## Conclusions

The adoption of the approach suggested by us will allow effective management of the pandemic, reduce unnecessary hospital admissions and expenditure, enable triaging of cases, and allow optimal engagement of healthcare professionals, thereby reducing the mortality and morbidity associated with the pandemic.

## References

[1] (2021). India. https://www.worldometers.info/coronavirus/country/india/.

[2] Kapoor G, Hauck S (2020). State-wise estimates of current hospital beds, intensive care unit (ICU) beds and ventilators in India: Are we prepared for a surge in COVID-19 hospitalizations?. medRxiv.

[3] Kim GU, Kim MJ, Ra SH, Lee J, Bae S, Jung J, Kim SH (2020). Clinical characteristics of asymptomatic and symptomatic patients with mild COVID-19. Clin Microbiol Infect.

[4] Park PG, Kim CH, Heo Y, Kim TS, Park CW, Kim CH (2020). Out-of-Hospital Cohort Treatment of Coronavirus Disease 2019 Patients with Mild Symptoms in Korea: an Experience from a Single Community Treatment Center. J Korean Med Sci.

[5] World Health Organization (2020). Home care for patients with suspected or confirmed COVID-19 and management of their contacts: interim guidance, 12 August. Home care for patients with suspected or confirmed COVID-19 and management of their contacts: interim guidance, 12 August.

